# Massachusetts Veterinary Association

**Published:** 1892-06

**Authors:** Austin Peters

**Affiliations:** Secretary


### SOCIETY PROCEEDINGS.

*Massachusetts Veterinary Association.*—The regular meeting of the Massachusetts Veterinary Association was held at 19 Boylston Place, Boston, Wednesday evening, March 23d. President L. H. Howard in the Chair. Members present, Drs. Bryden, Blackwood, Becket, Burr, Hadcock, Harrington, Howard, Lee, Marshall, Osgood, Skally, Winchester, Parker, and the Secretary. Honorary member, Dr. Stickney. Visitors, Dr La Baw and Messrs. Fowle, Murch and Maloney, senior students at the Harvard Veterinary School.

The records of the last meeting were read and accepted. Reports of committees: Executive Committee reports favorably upon the credentials of Dr. W. L. La Baw, presented at this meeting. Committee to report upon arrangments for the meeting of the United States Veterinary Medical Association, in Boston, next September, gave an account of what each member of the Committee present had done. The Chair then stated that a Commitee of arrangments from United States Association had been appointed, by President Huidekoper, with full powers to act; but it would be glad to receive any assistance or suggestions from members of the Massachusetts Veterinary Association's Committee or from individual members of our Association.

Quite a discussion then ensued upon the best place to hold a meeting, the entertainment our Association would offer the national body, and the best means of raising funds therefor.

New business. Dr. Lee as treasurer of a corporation asks for advice from the Association as to the proper means for the Boston Veterinary Hospital to pursue in regulating its charges. Competing with the Veterinary Department of Harvard University, it is found necessary in some instances to cut rates; that is, subscribers paying $10.00 a year to the Harvard Veterinary Hospital are entitled to having horses under treatment kept at $1.00 a day. The Boston Hospital charges $1.50 per day for each horse, but a number of the subscribers to the Harvard Veterinary Hospital who would patronize the new Hospital at $1.00 a day will not pay $1.50, so that it is necessary for the Boston Veterinary Hospital to take horses belonging to subscribers to the Harvard Veterinary Hospital at the same rates, that is, at $1.00 per day for each horse. After a short discussion Dr. Winchester rose to a point of order, asking the Chair if specific charges should not be brought in writing, if there were any charges to be brought up over which this Association had any ruling. The Chair ruled that if there are any charges to be brought forward they should be brought in a regular way.

Dr. Winchester moves that the Association hold its annual meeting at Young's Hotel. Also that a committee be appointed by the Chair to take charge of the details, a dinner to follow the meeting, which is called for 5.30 o'clock. Seconded by Dr. Osgood Carried.

The Secretary read a letter from Dr. W. T. Russell of Nashua, New Hampshire, asking if members could be admitted to the Association, who did not reside in Massachusetts. The Secretary was instructed to see the commissioner of corporations and find out if the Massachusetts Veterinary Association can legally admit non-resident members; if it can then he is to send Dr. Russell an application blank, in order that the Association can take action in the matter.

The Secretary then reported that Drs. Blackwood and Marshall had each attended more meetings during the year ending April 1891 than any other members, but that both had been to an equal number of meetings; which was to have Dr. Lee's prize tooth rasp? It was finally decided to consult their respective records of attendance for the year ending April 1892, and settle the matter in that way.

The Secretary then asked instructions as to whether the Association's accumulation of veterinary periodicals should be bound or not? Moved by Dr. Osgood, seconded by Dr. Bryden, that these magazines be bound. Carried.

Dr. Osgood moves: that this Association subscribe to McFadyeaus' Journal for this year, and that, if possible, the Secretary get the back numbers. Carried.

Dr. Howard then announced that the Committee to arrange for our annual meeting would be: Chairman, Dr. Winchester, with Drs. Lee and Osgood.

Dr. Marshall brought up the topic of death to animals by electric shock, and asked for views of the members present upon the subject. Very little information was elicited, until Dr. Winchester advised all interested to refer to the exhaustive report of the New York Board of Health upon the matter.

Meeting then adjourned.

(Signed) AUSTIN PETERS, *Secretary.*

